# Clinical Applications of FDG PET and PET/CT in Head and Neck Cancer

**DOI:** 10.1155/2009/208725

**Published:** 2009-08-20

**Authors:** Akram Al-Ibraheem, Andreas Buck, Bernd Joachim Krause, Klemens Scheidhauer, Markus Schwaiger

**Affiliations:** Department of Nuclear Medicine, Technische Universität München, Ismaninger Strasse 22, 81675 Munich, Germany

## Abstract

18F-FDG PET plays an increasing role in diagnosis and management planning of head and neck cancer. Hybrid PET/CT has promoted the field of molecular imaging in head and neck cancer. This modality is particular relevant in the head and neck region, given the complex anatomy and variable physiologic FDG uptake patterns. The vast majority of 18F-FDG PET and PET/CT applications in head and neck cancer related to head and neck squamous cell carcinoma. Clinical applications of 18F-FDG PET and PET/CT in head and neck cancer include diagnosis of distant metastases, identification of synchronous 2nd primaries, detection of carcinoma of unknown primary and detection of residual or recurrent disease. Emerging applications are precise delineation of the tumor volume for radiation treatment planning, monitoring treatment, and providing prognostic information. The clinical role of 18F-FDG PET/CT in N0 disease is limited which is in line with findings of other imaging modalities. MRI is usually used for T staging with an intense discussion concerning the preferable imaging modality for regional lymph node staging as PET/CT, MRI, and multi-slice spiral CT are all improving rapidly. Is this review, we summarize recent literature on 18F-FDG PET and PET/CT imaging of head and neck cancer.

## 1. Introduction

In 2008, head and neck cancers accounted for approximately 4% to 5% of all the malignant disease in the United States [1]. Head and neck squamous cell carcinoma (HNSCC) comprises the vast majority of head and neck cancer (HNC). Oncologic imaging plays an important role in head and neck cancers as imaging findings can aid significantly detection, staging, restaging, and therapy response assessment of these tumors. Accurate staging at the time of diagnosis is critical for selection of the appropriate treatment strategy. Unfortunately, at the time of initial diagnosis more than 50% of patients already present with regional nodal metastases or even distant metastases.

Diagnosis of a head and neck cancer is usually achieved by a combination of patient history, physical examination, and either nasopharyngoscopy and/or laryngoscopy with directed biopsies. Panendoscopy may be necessary to reveal the extent of a tumor. Morphologic imaging with computed tomography (CT) and/or magnetic resonance imaging (MRI) with intravenous contrast are often performed either prior to panendoscopy to noninvasively assess the aerodigestive tract or afterwards to provide information about primary tumor size, infiltration, involvement of surrounding structures, and regional nodal involvement. There is growing evidence, however, that these modalities have limitations in their diagnostic accuracy. CT and MR imaging rely on criteria of contrast-enhancement patterns and nodal size for detection of lymph node metastases which are not specific and may escape detection of metastases within normal size lymph nodes. There is also growing evidence that 18F-FDG PET imaging is a very sensitive and valuable imaging tool in evaluation head and neck cancer. The main drawback of 18F-FDG PET alone is the limitation with respect to lesion localization. However, the advent of PET/CT now overcomes this limitation and permits the evaluation of both metabolic and anatomic characteristics of disease, which has proven to be a major advance for staging, detection carcinoma of unknown primary, treatment monitoring, and evaluation of residual or recurrent disease.

## 2. Staging

Accurate staging at the time of diagnosis is the most important factor for treatment planning and determination of prognosis [8]. One attractive feature of 18F-FDG PET as a modality for initial TNM staging is that it covers most of the body within a single study. PET therefore provides information on the primary tumor, nodal metastases, distant metastases, and potential 2nd primary carcinomas. A literature survey on the use of 18F-FDG PET in head and neck cancer (HNC) compared to CT indicates that PET has a higher sensitivity (87% versus 62%) and specificity (89% versus 73%) for staging cancer [9]. Addition of PET/CT to initial staging of patients with HNC has also been shown to have a measurable impact on the treatment selection [10, 11]. 

### 2.1. Primary Tumor

Numerous reports on initial staging have shown that 18F-FDG PET is at least as sensitive as MRI or CT in detecting the primary tumor [3, 7, 10–17]. This is related to the fact that smaller or submucosal malignancies may be difficult to distinguish from adjacent tissues on anatomical imaging. A better sensitivity of 18F-FDG PET for detecting primary tumor comparing to CT/MRI imaging has been shown in oral cavity cancer [18, 19]. However, the current practice is not in favor of utilizing 18F-FDG PET for local staging of all newly diagnosed head and neck squamous cell carcinoma (HNSCC). This is due to the higher anatomic resolution of MRI and contrast enhanced multislice CT compared to 18F-FDG PET. Nevertheless, in a recent study by Baek et al. including 40 patients with oral cavity cancer and dental artifacts on CT or MRI, it was demonstrated that 18F-FDG PET/CT can provide more useful clinical information and higher sensitivity, particularly in deep tumors, compared to CT and MR. The diagnostic performance for the detection of the primary tumors in the oral cavity was 96.3% for PET/CT, 77.8% for CT, and 85.2% for MRI [20].

### 2.2. Nodal Metastases

Nodal staging has a significant impact on outcome in terms of disease free survival and overall survival after therapy [21]. Metastatic lymph node disease is found in approximately 50% of the patients at the time of primary diagnosis [6, 22]. Several reports have verified that 18F-FDG PET has a higher sensitivity and specificity than CT or MR imaging for detection of lymph node metastases in head and neck cancer [23, 24]. In a review by Schöder and Yeung, an average sensitivity of 87%–90% and a specificity of 80%–93% were reported for 18F-FDG PET/CT; a sensitivity of 61%–97% and specificity of 21%–100% were reported for morphologic imaging modalities including MRI and CT [25]. Several recent studies comparing 18F-FDG PET, 18F-FDG PET/CT, and CT/MR are summarized in Table 1. Results showed that integrated 18F-FDG PET/CT may play an important role in identifying lymph node metastases in head and neck squamous cell carcinoma (HNSCC). However, MRI is usually used for local staging as it provides almost comparable accuracy to 18F-FDG PET in locoregional metastases in addition to best primary tumor delineation [26].

Occult lymph nodes (clinical N0 disease) still represent a dilemma for both imaging modalities and surgeons. Although earlier reports have favored PET over other anatomic imaging modalities as PET has been shown to have a sensitivity of 78% and an accuracy of 92% (compared with a sensitivity of 57% and an accuracy of 76% for CT) for the detection of nodal metastases in clinical N0 disease [27]. Two recent reports by Nahmias et al. and Schoder et al. comprising 47 and 37 patients, respectively, demonstrated that 18F-FDG PET/CT is not accurate enough for detection of occult nodal disease in previously untreated patient and would not help the surgeon in the management strategy of the patient, particularly if the study is negative. They reported sensitivity and a specificity ranging from 67% to 79% and 82% to 95%, respectively. False negative findings were likely related to either the presence of microscopic metastases not detected by PET/CT, or by the proximity of nodal metastases to the primary tumor which might have obscured their detection [28, 29]. Schroeder et al. verified these results and suggested that elective neck dissection in patients with clinical N0 head and neck cancer squamous cell carcinoma (HNSCC ) should not be based upon cross-sectional imaging (CT, MR, PET/CT) at the resolutions currently available [30]. However, Kovacs et al. examined the potential role of 18F-FDG PET and sentinel node biopsy in 62 patients for the purpose of neck dissection. Their results suggest that patients showing positive lymph node on PET scan undergo a neck dissection due to the high specificity, whereas a sentinel node biopsy should be performed in patients with a negative PET scan. This strategy avoided 12 patients futile neck dissections with false-positive CT findings and a negative sentinel node biopsy [31].

### 2.3. Distant Staging

**Figure 1:**
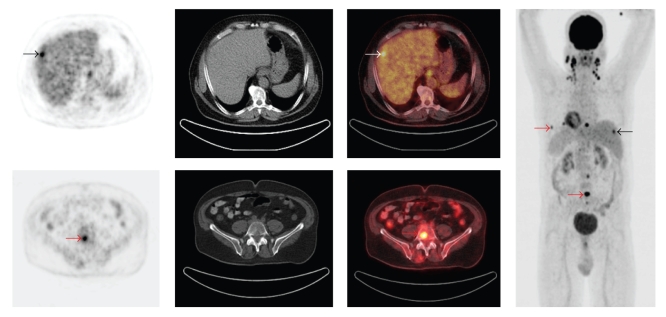
A 61-year-old man with nasopharyngeal SCC and bilateral cervical lymph node metastases underwent PET/CT for staging. Axial PET, CT, PET/CT, and maximum intensity projection (MIP) images are shown. PET/CT revealed focal FDG uptake in the right liver lobe indicating liver metastasis (black, white arrows). PET/CT also revealed multiple focal FDG uptakes in the lumbar spine, sternum, and ribs indicating multiple bone metastases (red arrows). PET/CT was valuable for detection distant metastases.

The role of 18F-FDG PET for staging of distant metastases in HNC is acknowledged as one of the most powerful indication in HNC (Figure 1). There is a general agreement that 18F-FDG PET is indicated for initial staging of HNC when there is suspicious of distant metastases and synchronous 2nd tumor. The incidence distant metastases increases with locally advanced disease (T3-T4), N2, or N3 disease, extracapsular extension of lymph node involvement, and perineural invasion [32, 33]. A synchronous 2nd tumor, particularly in aerodigestive tract, is often associated with a history of heavy nicotine or alcohol consumption and patients with hypopharyngeal tumors. Recent studies on the use of 18F-FDG PET for the detection of distant metastases and synchronous 2nd tumor in HNC are summarized in Table 2. These studies showed that PET detected distant metastases or 2nd primaries in up to 15.6% of the patients. The true positive findings were 82%. Moreover, PET showed a better accuracy once it was compared to conventional imaging as demonstrated by Ng et al., Chua et al., and Liu et al. [34–36].

## 3. Carcinoma of Unknown Primary

Cervical lymph node metastases from an unknown primary tumor account for approximately 1-2% of newly diagnosed head and neck cancers [40]. In 5% to 80%, depending on the patient selection, the primary tumor could not be identified by physical examination, panendoscopy, and conventional imaging, including CT and/or MRI [41, 42]. Treatment of these patients often includes extensive fields of irradiation to include the entire pharyngeal mucosa, larynx, and bilateral neck. The wide-field irradiation reduces the risk of tumor recurrence. However, it also causes significant morbidity, particularly in terms of xerostomia [43]. Therefore, the accurate identification of occult primary sites is important because the therapy can then be focused to the known site of origin, decreasing treatment-related morbidity, and improving therapeutic efficacy [44]. 

The utility of 18F-FDG PET to identify carcinomas of unknown primary has been examined. A comprehensive review by Rusthoven summarized the impact of 18F-FDG PET for the situation of carcinoma of unknown primary. A total of 16 studies comprising 302 patients, published between 1994 and 2003, were included. In all of these studies, patients underwent physical examination and CT or MRI, with the majority undergoing panendoscopy as well. The gold standard for primary tumor verification was tissue biopsy. Of the 302 patients, 18F-FDG PET detected 24.5% of tumors that were not apparent after conventional workup. 18F-FDG PET imaging also led to the detection of previously unknown metastases in 27.1% of the patients (regional, 15.9%; distant, 11.2%). The overall of sensitivity of PET for the primary tumor detection was 88%, with a specificity of 75%, and an accuracy of 79%. When detection efficacy was evaluated with respect to localization, a lower sensitivity for cancers in base of tongue, and a lower specificity for cancers in the tonsil were noted [45]. In this review, we performed a meta-analysis including studies published between 2000 and 2009 that specifically addressed the performance of 18F-FDG PET or PET/CT in detecting carcinoma of unknown primary in patients presented with cervical lymph node metastases and negative or inconclusive standard workup. For this group, 18F-FDG PET and PET/CT detected the primary tumor in 51 of 180 patients (28%) (Table 3).

**Figure 2:**
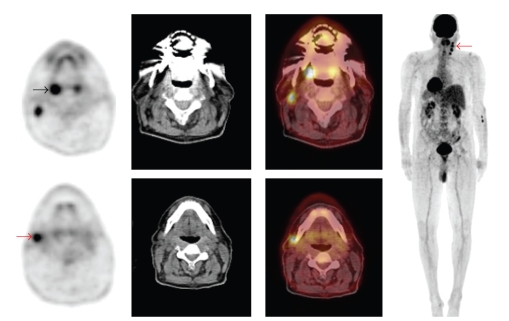
A 61-year-old man presented with right side cervical lymphadenopathy proved to be carcinoma of unknown primary. Patient underwent PET/CT to reveal primary tumor. Axial PET, CT, PET/CT, and maximum intensity projection (MIP) images are shown. PET/CT showed asymmetrical FDG uptake in the palatine tonsils with intense FDG uptake in the right tonsil (black arrow) as well as multiple hypermetabolic cervical lymph nodes in the right side (red arrows). This patient subsequently underwent surgical resection and histopathology revealed squamous cell carcinoma in the right tonsil. PET/CT was valuable in revealing the primary tumor in this case.

Two recent reports in the era of advanced morphologic imaging technology also verified the vital utility of 18F-FDG PET and PET/CT in cancer of unknown origin. Johansen et al. showed in a prospective study comprising 67 patients with cancer of unknown primary that a therapeutic change of treatment was made in 25% as a consequence of 18F-FDG PET findings [54]. Roh et al. compared the performance of combined 18F-FDG PET/CT and CT alone in 44 patients with cervical metastases from unknown primary tumors. They reported that 18F-FDG PET/CT was significantly more sensitive than CT (94.0% versus 71.6%, *P* < .001), but the two methods had similar specificities (94.8% versus 96.5%, resp.). 18F-FDG PET/CT correctly detected distant metastases in 6 out of 6 patients [55]. Based on these results, 18F-FDG PET and PET/CT can be recommended as early diagnostic modality in the workup for carcinoma of unknown primary and neck metastases (Figure 2).

## 4. Treatment Response Assessment

In recent years, the use of combined chemoradiotherapy (CRT) has been shown to have a significant impact on the treatment of head and neck cancer [56]. 18F-FDG PET is a valuable modality for monitoring response to treatment as it can assess metabolic activity rendering malignant process. Martin et al. demonstrated in a recent study including 78 patients that PET was significantly superior to clinical examination or conventional imaging with respect to the assessment of patients after chemoradiotherapy. Accuracy of PET in therapy response assessment was significantly better than clinical assessment and conventional imaging (CT/MR) (*P* < .002 and *P* < .001, resp.). The authors also suggested that patients with a complete response on posttreatment PET have a significant survival advantage [57].

**Figure 3:**
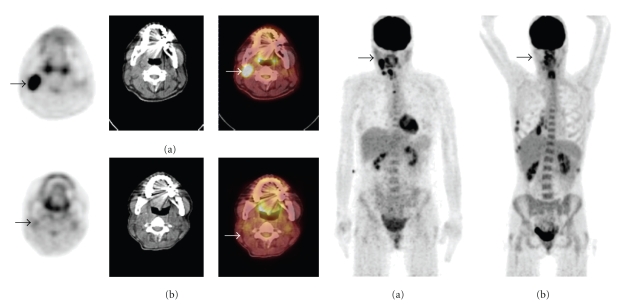
40-year-old women with right side larynx squamous cell carcinoma and a right side cervical lymph node metastases underwent PET/CT imaging before and 1 month after completion of chemoradiotherapy. (a) Pretherapy axial PET, CT, PET/CT, and MIP images reveal intense FDG uptake in the right cervical lymph node (arrows). (b) After treatment axial PET, CT, PET/CT, and MIP images reveal decrease FDG uptake in the corresponding locations (arrows). Appearance was consistent with early response to chemoradiotherapy. FDG PET/CT was valuable in monitoring early response to treatment.

Monitoring response to radiation therapy can be complex due to posttreatment changes like inflammation and edema. 18F-FDG PET has been investigated in the assessment of early response to chemotherapy regimen and if modification or discontinuation are needed or not (Figure 3). Several reports have illustrated that patients with favorable response to therapy demonstrate a continued reduction in metabolic activity and hence decreased FDG uptake over multiple PET studies compared to baseline values. Prognostic value of 18F-FDG PET regarding survival and response to therapy appears promising but needs more confirmation.

## 5. Residual and Recurrent Disease

The utility of anatomical imaging in the posttreatment situation is limited because of fibrosis, tissue edema, and anatomical distortion [58, 59]. The early detection of residual or recurrent head and neck cancer following radiation therapy and/or chemotherapy poses a diagnostic challenge. A survey of the literature showed that 18F-FDG PET is the most sensitive noninvasive modality presently available for differentiating posttreatment changes from residual or recurrent disease and that its performance is higher compared CT and MR for this purpose.

## 6. Residual Disease

A 3-4 months interval between the end of radiotherapy and evaluation of residual malignant tissue provides the best specificity and sensitivity for PET. This is due to reducing false positive findings associated with nonspecific inflammatory activity, and reducing false negative findings encountered during first 8 weeks postchemoradiotherapy which may increase the risk of seeding the dissection scar if viable tumor cell was left in the tumor bed [60–67]. The NPV of PET following therapy is very high (up to 97%) and associated with a very good prognosis, whereas positive 18F-FDG PET must be correlated with clinical status and a biopsy is needed to rule out nonspecific uptake [60, 61, 64–67]. Performance of 18F-FDG PET early after chemoradiotherapy has been evaluated to assess residual tumor as many surgeons prefer to perform salvage surgery within 6 to 8 weeks after radiation, before postradiation fibrotic changes develop. Kim et al. found in a prospective study in 97 patients that early imaging one month following completion of radiation therapy can be performed with high sensitivity (88%) and specificity (95%) [68]. Delbeke and Martin and Kostakoglu and Goldsmith agreed in two reviews that persistent tumor uptake one month after radiation therapy is strongly suggestive of residual disease and that a positive PET scan can result in immediate initiation of secondary treatment strategies due to early detection of resistance to chemotherapy [69, 70]. On the other hand, Rogers et al. found in a prospective study with a small number of patients (12 patients) a low sensitivity of 45% for a 1-month posttherapy 18F-FDG PET, compared to the 6–8 week posttreatment surgical histopathology [71]. The current role of 18F-FDG PET and PET/CT are indicated to exclude residual disease and to select patients who are candidates for salvage surgery after chemoradiotherapy. Although there is general consensus that waiting 3 months postradiation sustains best sensitivity and specificity, early imaging is justified in some scenarios, but with cautious interpretation considering time interval posttherapy and clinical findings.

The possible role of 18F-FDG PET in avoiding patients futile neck dissection after treatment by excluding residual locoregional disease has also been evaluated. Ong et al. demonstrated in a recent study comprising 65 patients that 18F-FDG PET/CT has a high negative predictive value (NPV) and specificity (97% and 89%, resp.) for excluding residual locoregional disease after chemoradiotherapy and that neck dissection may be omitted safely in patients without lymphadenopathy, while in patients with residual lymphadenopathy, a lack of abnormal ^18^F-FDG uptake in these nodes also excludes viable tumor with high certainty but still further assessment is needed [72]. Yao et al. suggested that 18F-FDG PET can avoid patient neck dissection if the postradiotherapy 18F-FDG PET scan is negative since it has had a high predictive value for negative pathology in neck dissection or fine-needle aspiration even with large residual lymphadenopathy [73]. Nevertheless, Tan et al. found in a retrospective study comprising 48 patients that 18F-FDG PET was not a good predictor of residual disease [74]. Standardization of the role of 18F-FDG PET in avoiding neck dissection in a prospective study particularly when lymphadenopathy presents is necessary before negative 18F-FDG PET/CT may become the only, or at least most-decisive, criterion in the management of the neck after chemoradiotherapy.

## 7. Recurrence

Klabbers et al. reviewed studies published between 1994 and early 2003 regarding the utility of 18F-FDG PET for detection of residual and recurrent head and neck tumors after radiation and/or chemoradiotherapy. The results showed that 18F-FDG PET has a better sensitivity (86%) and specificity (73%) compared with CT and/or MRI (56% sensitivity and 59% specificity, resp.) [75]. Ryan et al. reported on 108 patients and found that 18F-FDG PET/CT detected locoregionally persisting or recurrent head and neck SCC with a sensitivity of 82%, a specificity of 92%, a positive predictive value (PPV) of 64%, a negative predictive value (NPV) of 97%, and an overall accuracy of 90% [76].

18F-FDG PET and PET/CT have a high sensitivity and moderate specificity for detecting recurrent disease at the primary tumor site, regional lymph node metastases, and distant metastases. Wong performed a meta-analysis on studies published between 1999 and 2002 that showed relatively high sensitivity (84–100%) with moderate specificities (61–93%) regarding 18F-FDG PET in recurrent tumor of HNCSC [84]. Several studies published in the last 4 years on the utility of PET or 18F-FDG PET/CT for the detection of recurrence are summarized in Table 4. The performance of PET with respect to the identification of recurrent disease following treatment demonstrated a high sensitivity (83%–100%) and relatively high specificity (78%–95%). The higher specificity in these studies compared to an earlier report, published by Wong et al., may be related to more awareness of proper time point (2-3 months) for imaging after treatment.

## 8. PET/CT in Radiation Treatment Planning

New high-precision radiotherapy (RT) techniques, such as intensity-modulated radiation therapy (IMRT), 3-dimensional conformal radiotherapy (3D-CRT), and proton beam therapy allow conformal treatment of tumor and to avoid unacceptable damage to normal tissues leading to possible improvement of tumor control and decrease of treatment-related toxicity. These techniques depend on imaging modalities allowing accurate tumor volume delineation and response assessment during treatment. The potential application of 18F-FDG PET/CT for intensity modulated radiation therapy (IMRT) planning is an area of major interest. PET/CT may increase the gross target volume (GTV) because metabolically active tumor can be detected in normal-sized nodes. On the other hand, PET/CT-based target volume could be smaller than CT-based target volume alone in the case of patients in whom the tumor may be partially necrotic. The radiation treatment plan might be modified significantly if distant metastases are detected on the PET scan. The radiation target volumes may be significantly modified when 18F-FDG PET data are incorporated into radiation treatment planning. However, PET has been found to fail frequently to identify viable tumor in areas of bone marrow infiltration and perineural extension that are highly suspect on MRI (21).

Soto et al. suggested recently, based on a retrospective study comprising 61 patients, that 18F-FDG PET/CT should play an important but not exclusive role in defining the gross target volume (GTV) depending on the correlation between pretreatment 18F-FDG PET-defined biologic target volume (PET-BTV) and the anatomical sites of locoregional failure (LRF) after 3-D CRT or IMRT for head and neck cancer [85].

Rothschild et al. reported in a recent case control analysis of 45 patients with pharyngeal carcinoma that PET/CT and treatment with IMRT improved cure rates compared to patients undergoing IMRT without PET/CT. The event-free survival rate of the PET/CT-IMRT group was 90% and 80% at 1 and 2 years, respectively, compared to 72% and 56% in the control group without PET/CT (*P* = .005) [86].

Wang et al. investigated 28 patients with head and neck carcinoma treated with IMRT based on an 18F-FDG PET/CT defined gross tumor volume (GTV). They reported that tumor staging was significantly changed in 50% of cases (14/28 patients) as compared with CT-based staging, with 12 patients having higher T stages and 6 patients having higher N stages. Furthermore, a 17 months median follow-up period posttherapy did not reveal any locoregional recurrence indicating that PET-guided planning of the radiation field is accurate [87].

On the other hand, Breen et al. reported that GTV assessment in 10 patients with HNSCC was not significantly different between PET/CT and contrast CT scans, using 8 different observers. Furthermore, Breen et al. noted that there was greater consistency for the CT derived GTV's compared to the PET/CT derived volumes [88]. Table 5 summarizes recent studies on the use of 18F-FDG PET in radiotherapy planning. Ahn and Garg suggested in a review that the utility of a functional assay in defining target volume helps to determine areas to receive higher doses of radiation in cancers of the head and neck tumors [94].

One of the most controversial and challenging issues in applying PET/CT in radiation planning is contouring the outline of the tumor. Changing the PET window level can lead to a considerable overestimation or underestimation of the target volume. However, several techniques including threshold-based methods and gradient-based methods have been suggested and used, but still consensus needs to be met. Fifty percent of the tumor/image maximum intensity have been used for contouring by several groups [9, 95]. Others normalized volumes according to liver uptake [89, 91]. Wang et al. used an arbitrary SUV of 2.5 as a basis for contouring [87]. Berson et al. suggested in a recent report that developing an institutional contouring protocol for PET/CT treatment planning is highly recommended to reduce interobserver variability [96]. Geets et al. compared gradient-based method and threshold-based method in patients with laryngeal cancer. They demonstrated that the gradient-based method is more accurate than the threshold-based method. The threshold-based method overestimated the true volume by 68% (*P* = .014) [97].

Although most authors demonstrated that PET/CT can change the gross tumor volume (GTV) and staging status for radiotherapy planning. Several issues are still to be addressed before the role of PET/CT for IMRT planning and gross tumor volume (GTV) delineation can be clearly defined. Is this change in GTV clinically significant? Can PET/CT provide prognostic information guiding the escalating of the radiation dose to area with higher metabolic activity? Furthermore, is the development of objective and reproducible methods for segmenting PET images achievable? Addressing these issues will help to identify the ultimate impact of this technology in radiation treatment planning which needs subsequent larger experimental studies with clinical outcome and cost-benefit analyses.

## 9. Non-FDG PET in Head and Neck Cancer

PET imaging has become a promising tool for detecting hypoxic subvolumes of tumors. Hypoxia represents a negative prognostic factor for radiation treatment of head and neck cancer where it is associated with a significant resistance to radiochemotherapy [98, 99]. However, mapping hypoxic region of tumor can positively impact on treatment outcome [100]. Several PET tracers have emerged for this purpose like 18F-fluoromisonidazole (18F-FMISO), 18F-labelled fluoroazomycin arabinoside (-[18F]FAZA and 2-(2-nitro-(1)H-imidazol-1-yl)-N-(2,2,3,3,3-pentafluoropropyl)-acetamide (EF5) [100–102]. Chao et al. demonstrated the feasibility of Cu(II)-diacetyl-bis(N(4)-methylthiosemicarbazone) (60Cu-ATSM PET) to create a hypoxia imaging-guided IMRT treatment plan through coregistering hypoxia 60Cu-ATSM PET to the corresponding CT images for radiation therapy of patients with head and neck cancer [103]. At our institution, Souvatzoglou et al. 18F-labelled fluoroazomycin arabinoside (18F-FAZA) as a feasible hypoxic agent in patients with head and neck cancer and demonstrated that FAZA can be potentially used for hypoxia-directed intensity-modulated IMRT dosing patients [104].

While 18-18F-FDG PET is very sensitive head and neck cancers, its specificity is not as high as its sensitivity due to false-positive results in inflammatory or infectious lesions. These lesions are frequent in this area, in particular after treatment by surgery and/or radiotherapy. For this purpose, O-(2-[18F]fluoroethyl)-L-tyrosine (18F-FET) has been introduced and investigated by several groups. Results suggested a possible role for FET in head and neck cancer to differentiate between inflammatory and malignancy in a selective cases. Nevertheless, it should not be used as alternative to FDG due to inferior sensitivity [105, 106].

The proliferation marker fluorodeoxythymidine 18F-3-deoxy-3-fluorothymidine (18F-FLT) has been investigated by de Langen et al. in 15 patients (including 6 patients with HNC) to evaluate the reproducibility of quantitative 18F-FLT measurements. The authors showed that quantitative 18F-FLT measurements are reproducible for predicting response to therapy in individual patients. However, authors recommended further studies correlating 18F-FLT response with pathological and clinical outcome [107].

**Figure 4:**
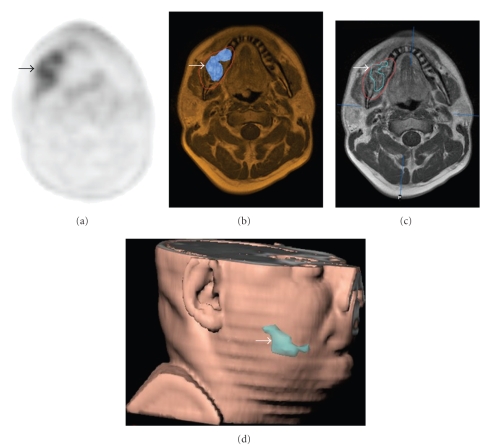
Patient with a squamous cell carcinoma of the right mandible (arrows). (a) The [18F]Galacto-RGD PET shows heterogeneous tracer uptake, which can also be clearly delineated in (b) the image fusion with the corresponding MRI scan. (c) shows the tumour volume in red as defined by MRI. By applying a threshold of SUV 3 and only using pixels with SUVs above this threshold, (d) a subvolume with more intense *α*vß3 expression can be defined which is shown in the 3D reconstruction (blue line in (c), blue area in (d)).

Beer et al. investigated the application of [18F] Galacto-RGD-PET imaging of *α*vß3 expression, a receptor related to tumor angiogenesis and metastasis, in 11 patients with head and neck squamous cell carcinoma (HNSCC). Their results showed that use of 18F-RGD PET in a multimodalities setting and definition of tumor subvolumes is feasible (Figure 4). The authors suggested that 18F-RGD PET imaging might be used for the assessment of angiogenesis and for planning and response evaluation of *α*vß3 targeted therapies [108].

In the preclinical settings, the role of molecular imaging with PET for monitoring the antiepidermal growth factor receptor (anti-EGFR) inhibitor therapy in solid tumors showing overexpression of EGFR like head and neck squmous cell carcinoma (HNSCC) has been investigated. Several radiopharmaceuticals including the proliferation marker fluorodeoxythymidine (18F-FLT) and the chimeric monoclonal antibody (64CU-DOTA-Cetuximab) have been considered promising for this purpose. However, further clinical and imaging studies are still needed.

## Figures and Tables

**Table 1:** Studies comparing accuracy of FDG PET and PET/CT with CT and MRI for detection of lymph nodes metastases.

Author year	Number of patients	Tumor Subtypes	Results	Notes
Beak et al. [2], 2009	15	Periorbital	PET/CT accuracy (98%) > CT 84%	- CT: 16 slice. - PET modified Tx in 39%.
Roh et al. [3], 2007	167	HNSCC	PET or PET/CT accuracy (92%-93%) > CT/MR 85%-86%	- PET/CT significantly better for detection of primary tumor
Gordin et al. [4], 2007	35	Nasopharyngeal	PET/CT accuracy 91% > PET 80% > CT 60%	- Retrospective - PET/CT modified TX in 57%
Kim et al. [5], 2007	32	Oropharyngeal	PET sensitivity 21% higher than CT/MR (*P* < .05)	- PET/CT significantly better for detection of primary tumor
Dammann et al. [6], 2005	79	Oral cavity and oropharynx	PET accuracy 96% > MRI 94% > CT 92%	- Nonhyprid PET/CT used
Ng et al. [7], 2005	124	Oral cavity SCC	PET accuracy 98.4% > CT/MR 87.1%	- Prospective

**Table 2:** Studies evaluating the performance of FDG PET for the detection of distant metastases and synchronous 2nd tumor in HNC.

Author year	Number of patients	Positive PET	True positive (distant mets + 2nd primary)	False positive	Notes
Ng et al. [34], 2009	111	16	13/16	3/16	CT/MR detect 4/16
Chua et al. [35], 2009	68	6	5/6	1/6	CT + BS detect 4/6
Liu et al. [36], 2007	300	61	50/61	11/61	
Yen et al. [37], 2005	118	32	24/32	8/32	
Goerres et al. [12], 2003	34	8	7/8	1/8	PET modified Treatment in 15%
Sigg et al. [38], 2003	58	8	7/8	1/8	PET modified Treatment in 5%
Schwartz et al. [39], 2003	33	7	7/7	0/7	

Total	722	138	113/138	25/138	

**Table 3:** Studies evaluating performance of 18F-FDG PET or PET/CT for the detection of carcinoma of unknown primary in patients with negative workup.

Author year	Number of patients	All positive	True positive	False positive	Percent detected by PET
Padovani et al. [46], 2009	13	9	7	2	54%
Silva et al. [47], 2007	25	9	3	6	12%
Fakhry et al. [48], 2006	20	10	7	3	35%
Wong and Saunders [49], 2003	17	8	5	3	29%
Fogarty et al. [50], 2003	21	6	1	5	5%
Johansen et al. [51], 2002	42	20	10	10	24%
Kresnik et al. [52], 2001	15	12	11	1	73%
Jungehulsing et al. [53], 2000	27	7	7	0	26%

Total	180	81	51	30	28%

**Table 4:** Studies evaluating the performance of 18F-FDG PET and PET/CT for the detection of recurrent disease in head and neck cancers.

Authors year	Number of patients	Sensitivity	Specificity	Accuracy	Notes
Abgral et al. [77], 2009	91	100%	85%	90%	FDG PET/CT
Wang et al. [78],2009	44	100%	98%	98%	Prospecrive PET performance > CT
Cermik et al. [79], 2007	50	83%	93%		
Álvarez Pérez et al. [80], 2007	60	98%	90%		Prospective
Salaun et al. [81], 2007	30	100%	95%	97%	
Goerres et al. [82], 2004	26	91%	93%		Prospective
Kubota et al. [83], 2004	36	90%	78%	81%	Prospective Accuracy significantly higher than CT/MR

**Table 5:** Studies evaluating the role of FDG PET and PET/CT in radiation planning.

Author year	Number of patients	Study type	Results	Notes
Soto et al. [85], 2008	61 (9 LRF)	Retrospective	8/9 LRF within BTV-PET.	
Rothschild et al. [86], 2007	45	Case-control analysis	PET/CT with IMRT improved cure rates	Advanced pharyngeal carcinoma
Wang et al. [87], 2006	28	Prospective	PET/CT-based GTV significantly different from CT scans alone in 50% of cases	PET/CT upgraded T and N stage in 18 p.
Breen et al. [88], 2007	10		no significant differences in the GTVs between PET/CT and CT alone	CT volumes were larger than PET-CT
El-Bassiouni et al. [89], 2007	25		PET/CT-based volume significantly smaller than CT.	
Koshy et al. [90], 2005	36	Retrospective	TNM changed in 36%, RT volume and dose changed in 14%	
Heron et al. [91], 2004	21	Prospective	PET/CT improves delineation of normal tissues from tumor areas	PET/CT improves staging
Ciernik et al. [92], 2003	12HNC of 39	Retrospective	PET/CT changed GTV in 50% compared to CT	
Nishioka et al. [93], 2002	21		PET improves GTV, normal tissue sparing	PET alone

(IMRT) intensity-modulated radiation therapy, (GTV) gross target volume, (BTV) biological target volume, (LRF) locoregional failure.
